# Swedenborg's Dreams

**Published:** 1861-10

**Authors:** 


### Art. VI.—SWEDENBORG'S DREAMS.

In the whole range of modern biography there is no life of greater interest to the medico-psychologist than that of Emanuel Swedenborg. His writings constitute a splendid monument of the extraordinary intellectual powers, the untiring assiduity, and (apart from all considerations of the ultimate results to which this fervour led him) the lofty religious fervour of the man. As a philosopher he will always occupy a conspicuous and honourable position in the history of modern philosophy,† and as a theologian he gave birth to one of the most remarkable developments of Christianity in recent times. From the beginning, his religious feelings entered largely into his philosophical speculations, and constituted an essential portion of them. He held, indeed, that religion was necessary to the perfection of philosophy, and that philosophy divorced from religion was a dead letter. With him philosophy and revelation were fundamentally one and the same.

The merits of Swedenborg as a philosopher are too apt, perhaps, to be overlooked at a time when, as in the present day, his

---

† Writing of Swedenborg's philosophy, Morell remarks that it is "perhaps the only attempt the world has seen (with the exception of the unsuccessful effort of Comte) as rising upwards to purely philosophical ideas from positive and concrete facts."—*History of Modern Philosophy,* vol. ii. p. 321.

opinions are known chiefly through the medium of those works of his for which he claimed the authority of divine revelation. "I have," he wrote in 1769, "been called to a holy office by the Lord Himself, who most graciously manifested Himself in person to me, His servant, in the year 1743, and then opened my sight into the spiritual world, and endowed me with the gift of conversing with spirits and angels, which has been continued to me to this day."*

This claim is by no means so obvious that it could be received without strong confirmatory evidence. Now many think that this is afforded by the intrinsic character of the so-called revelations made to Swedenborg and recorded by him, and their faith in this view is sufficiently evidenced by the existence of a distinct branch of the Christian Church, founded upon the doctrines taught by him. On the other hand, it is contended that there is nothing in the evidence derived from the supposed divinely illumined writings of Swedenborg which might not have pre-existed in his mind, and which is not consistent with, and a possible consequence of, his habits of thought. Hence it is argued, that the hypothesis of spiritual prompting is neither necessary nor probable, and that Swedenborg had, unfortunately, in common with many mystics, become subject to hallucinations which were characteristic of his dominant ideas and feelings. This is the simplest and most natural conclusion, and it derives strong confirmation, if it is not actually demonstrated, by a work of rare interest which has recently come to light.

For the subsequent account of this work, and extracts from it, we are indebted to Dr. William Daniel Moore, of Dublin, M.R.I.A., Honorary Member of the Swedish and Norwegian Medical Societies, to whose distinguished talents as a linguist the medical profession of this country has been so often and so greatly indebted.

In 1858, there was discovered among the papers of a Professor Scheringsson, of Westerås, who had died in 1849, and among whose literary remains the document had been overlooked for nearly ten years, a singularly interesting and previously unknown manuscript of Swedenborg's. In what manner this manuscript had come into the possession of Professor Scheringsson, how long it had been in his hands, and what motives could have induced him to keep its existence a profound secret, are questions concerning which no information has been ascertained. No doubt would appear to be entertained as to the genuineness of the manuscript, which is a species of "Spiritual Diary" of the author for the year 1744, written in old Swedish, and it was

* Answer to a letter from a Friend, prefacing *A Treatise concerning Heaven and its Wonders, and also concerning Hell.* Lond., 1817.

purchased for, and is now deposited in, the Royal Library of Stockholm.

In 1860 this "Diary" was published under the following title—*Swedenborg's Drömmar* 1744 *jemte andra hans anteckningar. Efter original-handskrifter.* Ita provisum est a Domino, ut phantasiæ iis appareant prorsus sicut realiter forent.—*Diarium Spirituale*, 4360 (Stockholm, J. och A. Riis. 1860. 8vo., pp. 94, Swedenborg's Dreams, 1744, with other notes of the Author. From original MSS.). Appended to the pamphlet is an introduction by the Editors, entitled, "*Reflexioner öfver de nyligen uppdagade Swedenborg's Drömmar* 1744" (Reflections on the recently discovered Dreams of Swedenborg, 1744, pp. xxiv.).

The "Dreams" would appear to have been noted down for the purpose of refreshing the author's memory as to his past psychical and spiritual condition, and they were most probably intended for no other eye than his own. For convenience of description the dreams recorded may be divided into six categories—

1. The *common-place*, comprising by far the greater number. From many of these, although they differ but little, if at all, in character from the strange ideas which float through the minds of most people in their sleeping moments, the author derives indications of his own sinfulness and helplessness, of the mercy of God exhibited towards him, of forgiveness of sins, and acceptance with the Deity.

2. The *amatory*. Some of this class are lascivious and obscene. They are generally followed by expressions of repentance and of a "sense of forgiveness of sins," &c.

3. The *horrible*, or those characterized by hideous apparitions. Several of these are evidently of the nature of nightmare.

4. The *ambitious*, or those in which the dreamer converses with kings and emperors.

5. The *penitential* or *devotional*.

6. The *ecstatical*, or those in which the author supposes he has been favoured with manifestations of the Divine presence.

Before citing any illustrations of the 'dreams,' it is necessary to premise that it is by no means an easy task at all times to ascertain the precise meaning of the old Swedish in which Swedenborg has noted them. Not only does the language itself appear to have undergone a considerable change within the past century, but an additional source of obscurity is found, in the mystical signification attached to the facts recorded. "A certain class of so-called cultivated people," say the editors, and, it must be added, apologists of the "Dreams," in explanation of the somewhat corrupt language in which they are written, and of their occasional obscenity, "little knows what change the Swedish tongue has undergone within the last century, and still less do such people understand the imagery, the sublimely symbolical forms through which a spiritual world, by means of dreams and visions, under certain circumstances and for definite objects, reveals itself and condescends to a natural world." When a sentence is doubtful, or, so far as we can discover, unmeaning, we have placed it within brackets.

The first detailed dream bears date 24 × 25* March, 1744: previously to this are some notes commencing on the day of Swedenborg's departure from Stockholm, the 21st July, 1743. These notes contain certain heads of dreams apparently taken down before Swedenborg began to write out his dreams at length, and they run thus—

"1. in youth and the Gustavian family.

"2. in Venice about the beautiful palace.

"3. in Sweden, about heaven's white cloud.

"4. in Leipsic, about him who lay in boiling hot water.

"5. about him who tumbled down with the chain into the abyss.

"6. about the king, who gave so richly in a cottager's hut.

"7. about the servant who wished I should depart.

"8. about my enjoyments in the night.

"— wondered in myself that I had nothing remaining to do for my own honour, so that I found feeling thereof.

"— [that nothing was borne (? portrad) before the sex, I had been all my days.]

"9. how I had been in extasibus vigilibus almost the whole time.

"10. how I set myself against the spirit.

"—and how I then thought thereon, but found afterwards that it was folly, without life and connexion.

"—[and that thus a quantity in what I have written must be, as I have not in the degree forsaken the spirit's power, for the faults are mine own, but the truths are not mine.]

"— I sometimes got impatient and thought, that I seemed as though I would presume when it went not so easy as I wished, after I did nothing for mine own sake; found my unworthiness less, and thanked for grace.

"11. how I found that after I came to the Hague the propensity and self-love of my work was passed away, this I myself wondered at.

"— how my inclination for woman so hastily terminated, which had been my ruling passion [hufvudpassion.]

"— how I had the whole time the best sleep at night, which was more than kind.

"— how my ecstasies before and after sleep.

"— my clear thoughts on matters.

"How I set myself against the Holy Spirit's power, and what

* This mark ( × ) interposed between the dates signifies *night*.

happened thereupon, how I saw hideous apparitions; without life, horribly involved, and therein had to do with an animal which attacked me and not the child.

"I seemed to lie on a hill, under which was an abyss; there were knobs; I lay there, would help myself up, was held in a knob, without foothold; an abyss was under me; signifies that I would help myself from the abyss, which was not possible.

"How a woman lay down at my side, so when I was awake I would know what it was; she spoke gently, but said she is pure; but I smell ill, which was my guardian angel, as I believe, for then the temptation began!"

Of the detailed dreams, as well as of the psychical condition of Swedenborg at the time when they occurred, the following quotations, we trust, will convey a tolerably accurate conception:—

1744, March 24 × 25—"Spoke with our successor in Sweden, who was turned into a woman, yet was also familiar, afterwards with Carl Brokman. I know not what this signifies if not from the following.

"Came into a magnificent chamber, and spoke with a woman, who was the governess; she would inform me of something; then came the queen in and passed through into another chamber; she seemed to have been the same who represented our successor. I went out, for I was very meanly clad—having come from my journey—a long old surtout, without hat or periwig, I wondered that she deigned to come after me; she told me that one had given her mistress all the jewels, but she got them back by saying that he had not given the best; then she threw away the jewels; she told me to go in again, but I excused myself because I was so ill-clad, and had no periwig, I should first go home; she said it did not signify: it concerns that I should then write and begin the epilogue of the second part, [this I would sit down to, but it was not necessary; which also happened; what she stated about the jewels, are truths which are disclosed, but have been taken back, because she was angry not to get all; I afterwards saw the jewels in her hands, and a great ruby among them.]" (p. 5.)

"5 × 6, April, 1744—Easter-day was the 5th April: when I went to God's table, the temptation still continued, most of the afternoon till six o'clock, yet nothing certain; it was an anguish as if one were condemned, and in hell, yet the hope, which the Holy Spirit gave, was always strong, as Paul says, Rom. v. 5, the devil was given power with various thoughts to make the inner man uneasy. Whitsunday after Communion I was inwardly cheerful, but still outwardly sad; the temptation came in the afternoon, in a quite different manner, but strong; though I was assured that I had obtained forgiveness of sins, still I could not direct my flying thoughts, nor avoid expressing something against my better reason, which was the devil's, through permission; prayer alleviated them, as also God's word, faith was there intact, but confidence and love seemed to be away. I lay down at 9, the temptation continued with trembling until half-past 10 o'clock; then I fell into a sleep, in which all my temptation was represented, how * * * * sought in various modes to get me on his side, to be of his party (luxury, riches, vanity), but he could not. I was still more obstinate on the subject, when he manifested disdain towards me; afterwards I was together with a serpent, dark grey, which lay, and was B.'s dog. I struck many blows with a club after him, could never hit him on the head; it was fruitless; he would bite me, but could not; I took him by his mouth, he could not bite me, nor could I do him much harm; at length I took him by the jaws, and he squeezed hard, and by the nose; I squeezed it that it broke out, like poison; said, that the dog did not belong to me, yet, as he would bite me, I must chastise him; thereupon he seemed to say that he had not got me to say a word with him, I wrangled so with him. When I awoke, the word I said was, Hold thy tongue; hence may be seen, without further explanation, of what nature the temptation was; but on the other hand, how great God's grace was, through the merit of Christ, and the operation of the Holy Spirit, to which be glory for ever and ever. I immediately thought how great the Lord's grace is, which imputes to us that we resist temptation, which nevertheless is only God's grace and operation, is his, and not ours, and overlooks what infirmities we have had therewith, which yet must be manifold, as also what great glory our Lord gives, after a short period of adversity." (pp. 8, 9.)

Swedenborg's "first revelation," according to the "Dreams," occurred at the Hague, 6 × 7 April, 1744, and not in 1743, as stated in the letter quoted at the beginning of this article, and is thus described:—

"In the evening I fell into another sort of temptation, namely, between 8 and 9 o'clock, when I read God's miracles, performed through Moses, I thought that somewhat of my understanding was mixed up therewith, that I could not have the strong faith I ought; I believed and did not believe, thought that therefore God and the angels manifested themselves to shepherds and not to the philosopher who appeals to his reason, asking why God made use of the wind to bring up the locusts, why he hardened Pharaoh. I looked at the fire, and said within myself, so should I also not believe that the fire exists, as the outward senses are more fallacious than what God says, who is ipsa veritas, I ought to believe this rather than myself. With many such thoughts I passed an hour or an hour and a half, and smiled in my mind at the tempter. It is to be observed that the same day I went to Delft, and the whole day had the grace to be in deep spiritual thought, as deep and beautiful as I ever had, and the whole day, which was the Spirit's work.

"At 10 o'clock I lay down in bed, and was somewhat better; half an hour after I heard a clamour under my head, I thought that then the tempter went away: immediately there came over me a rigor so strong from the head and the whole body, with some din, and this several times; I found that something holy was over me; I thereupon fell asleep, and at about 12, 1 or 2 o'clock in the night, there came over me so strong a shivering from head to foot, with a din, as if many winds rushed together, which shook me, was indescribable, and prostrated me upon my face. Then while I was prostrated I was in a moment quite awake, and saw that I was cast down, and wondered what it meant. And I spoke as if I was awake, but found that the word was put into my mouth, and I [said], Omnipotent Jesus Christ, as of thy great grace Thou condescendest to come to so great a sinner, make me worthy of this grace. I held my hands together and prayed, and then came a hand, which squeezed my hands hard; immediately thereupon I continued my prayer, and said, that Thou hast promised to pardon all sinners, Thou canst not but keep thy word; at the same time I sat in his lap and saw him face to face; it was a face of holy look, such as cannot be described, and smiling, such as I believe his face was while He lived. He spoke to me, and asked whether I had a bill of health; I answered, Lord, That thou knowest better than I. Well, do so, said he. That is, I thought, Love me really, or do what thou hast promised. God give me the grace thereto. I found that it depended not on my own strength, woke with rigors: I again came into such a state that I was, in thought, neither sleeping nor waking. I thought, what can it be? Is it Christ, God's Son I have seen? but it is sin to doubt it. But as it is commanded that we shall try the spirits, I thought, after all, and found from what passed the night before, that I was purified and preserved, and so prepared for it, as also that I fell upon my face, and the word I spoke, and the prayer came not of myself, but the word was put into my mouth, yet that I spoke, and that all was holy: so that I found that it was God's Son himself, who came down with such a din, and who prostrated me on the ground of himself, and made the prayer, and so said it was Jesus himself. I prayed for pardon, that I should so long have doubted it, and also that it came into my thought to desire a miracle; this I found was improper. Thereupon I fell to prayer, and prayed only for pardon; more I could not, but afterwards I added, and prayed that I might obtain love, which is Jesus Christ's work and not mine. However, rigors often passed over me." (p. 11.)

"To forget nothing, it also came into my thoughts that the Holy Spirit would show me to Jesus, and present me to Him as a work He has so prepared, and that I ought not to attribute anything to myself, but all is his, although He, of grace, attributes the same to us.

"So I sang the psalm I then chose; Jesus is my best Friend, n. 245.

"This I have now learned in spiritual matters, that there is nothing else than to humble one's self, and not to desire anything else, and that with all humility, than the grace of Christ; I added of myself, to obtain love; but this is arrogant, for when one has God's grace he places himself in Christ's hands, and does according to his will: man is happiest when he is in God's grace; I had with most humble prayer to supplicate for forgiveness before my conscience could be in peace, for I was still in temptation before that took place; the Holy Spirit taught me this, but in my stupidity I overlooked humility, which is the foundation of all." (p. 13.)

"April 15 × 16.—I thought I climbed by a ladder out of a great abyss; after me came other women whom I knew; I stood still and frightened them on purpose, went up, struck against a green sward, and lay down: the others came after I saluted them, they were women, they lay down beside me, one young and one a little older. I kissed the hands of both, and knew not which I should love. (p. 29.)

After the foregoing, April 17 × 18 brings its "horrible dreams, how the executioner spitted the heads he cut off, and laid one after the other in an empty oven which never became full: he said it was his food; he (sic) was a large woman, mean, had a little girl with him [or her.]

"Afterwards how the Devil led me into several abysses and bound me, I remember not all. I was cast everywhere bound into hell.

"How a great procession was formed, from which I was excluded; and that I should have come from it; but I endeavoured to come thither, sat down there, but they advised me to go from it. I went; yet I had another room to see it from, which had not yet come. Yet am I certain that God shows mercy and pity to all poor sinners, who will repent, and with steadfast faith fly to his incomprehensible compassion, and to the merits of our Saviour Jesus Christ. So I feel assured of his grace, and leave myself in his protection, because I believe certainly that I have obtained forgiveness of sins; which is my consolation, which may God, for Jesus Christ's sake, strengthen." (p. 31.)

"I obtained peace, God strengthen me therein, for it his work, and so much the less mine, as my thoughts, even the best of them, rather disturb than promote it. Then one smiles in himself, both when he thinks contrary to it, as when he will confirm with his understanding what he believes. Therefore it is a higher degree, I know not if it is the highest, when a man acquires grace, not to mix up his understanding in matters of faith, (although it would appear that our Lord in certain cases admits that reflexion is to be preferred to authority in what concerns the understanding;) 'blessed are they who believe and have not seen.' This I have clearly written in prologue n. 21, 22; but yet could not of myself remember or arrive at it, unless God's grace, without my consciousness, had effected it, as I subsequently found from the effect itself and the change in all my inner being, for it is God's grace and operation, to which be glory for ever; for I hence see how difficult it is for the learned, compared with the unlearned, to arrive at this faith, and so to overcome themselves, as to smile at themselves; for admiration of one's own understanding must first of all be subdued and cast down; which is God's work and not man's. Likewise is it God's work to maintain one therein. This faith is therefore distinct from our understanding, and is above it. This is pure faith, the other is impure, so long as it is mixed up with our understanding; we ought to take our understanding captive under obedience to faith. That we ought to believe therefore, hath he said who is one God over all, truth itself. This is what seems to be meant by the saying, that we ought to be as children. Much of what I have seen agrees herewith, and perhaps this also, that so many heads were cut off and cast into the oven, which was the Devil's food."

"And that confirmatives darken faith, is seen from the fact that the understanding does not reach beyond probabilities, wherein there is always as it were *probatio majoris* or *minoris*, for confirmatives of one's own understanding are always subject to doubt, which obscures the light of faith. This faith is solely God's gift, which one obtains, if he lives according to God's commandments, and assiduously prays for it." (p. 32.)

Speaking in another place of conflict with temptation, he says, "I may liken it to a pair of scales, in one lie our will and evil nature, in the other God's power, which our Lord arranges so in temptation, that he allows it sometimes to come to an equilibrium, but so soon as it is about to weigh down to the side, he helps it up; thus I speak in worldly language, whence it follows that our power is so small that it drags all down and is rather opposite than auxiliary to the Spirit's power; and thus that it is only our Lord's work which he so arranges." (p. 15.)

Though a great dreamer, Swedenborg seems to have been a good sleeper, for he tells us that he "slept for an hour and an half, although during the night I had slept for more than ten hours. I have by God's grace had supernatural sleep, as also during the whole half year."

"I held my hands together; on awaking I thought they were pressed together by a hand or finger, which with God's help signifies that our Lord heard my prayers."

"This day I was in the strongest temptation, so that when I thought upon Jesus Christ godless thoughts forthwith came into my mind, which I could not get the better of: in my opinion, I beat myself, but can acknowledge that I never was in so healthy a mood as this day, and was not in the least timorous or grieved as the other days, although the temptation was strongest, because our Lord gave me the firm belief and confidence that he helps me for Jesus Christ's sake and his promise, so that I then experienced what the efficacy of faith is." (p. 34.)

But, alas! the flesh soon again gets the better of the spirit, for the private records of 23 × 24 April in Leyden, and of 28 × 29 at the Hague, bear so strong a resemblance to some objectionable passages in Horace and Juvenal, that we must omit much of them.

"23 × 24 [April, 1744], in Leyden. It seemed that I was fighting with a woman in flight, who drove me into the sea and up again; at length I struck her with the plate on the forehead, as hard as could be, and pinched her face, so that she seemed to be overcome; it was my afflictions and my contest with my thoughts which I overcame.

"— It seemed to be said, interiorescit, integratur, which signifies that I am inwardly cleansed by my afflictions.

"— Afterwards something holy was dictated to me the entire night, which ended with sacrarium et sanctuarium: I found myself lying in bed with one [a female], who said, If thou hadst not said sanctuarium, we should do." [The sentence following is highly obscene.]

"There was one waiting at the bedside, but she went away first.

"This signifies the most extreme love for sanctity, for all love has its origin thence, is a series; in the body it is actually in projectione seminis, when the whole seed (saten [?]) is there and pure, it signifies the love for wisdom. The former was for truth; yet as there was one listening thereto and nothing was done until she was gone away, it signifies that silence ought to be observed respecting it, and none to hear of it, for to the worldly understanding it is impure, though of itself pure.

"Afterwards I slept a little, and it seemed to me that oil mixed with mustard still flowed, which seems to be my future life, and which shows there is enjoyment mixed with some adversity, or that it signifies a medicine for me.

"This took place at Leyden, in the morning of the 24th of April.

"28 × 29 [April, 1744.] The night before I thought I saw King

Charles XII., to whom I formerly dedicated my book; but I thought now that he was risen from the dead, and that I went out, and would now dedicate to him, as it were, another.

"I went by a way, it was a cross road I was directed to go up, so I went, but thought there were only some days left, so I went back into the plain; there was much people, I would go out, and was very much pressed.

"I gave some fruits to a gardener to sell; he sold them, and brought me back two Carolines, but said that he had kept for himself thirteen dollars; this I did not care for." [The sentence following is most filthy and obscene.]

"All this shows, I think, that I ought to employ my remaining time on what is higher, and not in writing on worldly things, which are far inferior, but on what concerns the very centre of all, and what relates to Christ. God be so gracious and instruct me further what my duty is, for I am still in some darkness as to whither I ought to turn myself.

"d. 1 × 2 July," describing a manifestation he says: "This was in a vision, when I was neither awake nor asleep, for I had all my thoughts collected; *it was the inner man separated from the outer*, which perceived it: when I was quite awake, similar shiverings came several times over me. This must have been a holy angel, for I was not cast upon my face; what that signifies, our Lord knows best; it seemed before to be said to me, that I should have something for my obedience or something else. God's grace was manifested to the inner and the outer man in me; to God alone be praise and glory."

6 × 7 October, he observes, "I thought and perceived that all love to anything whatever, as to the work I have in hand, if we love it in itself, and not merely as a medium to the only true love which is to God and Jesus Christ, is a meretricious love, and is therefore always likened to whoredom in God's word; this is also what has happened to myself; but when our love to God occupies the first place, we have no other love besides than what promotes our love to God." (p. 55.)

The foregoing extracts will probably suffice to convey a pretty accurate notion of Swedenborg's psychical state, at the time when his visions commenced. It would seem that in 1743—1744, Swedenborg had become subject to frequent dreams, contemporaneously with a marked, and, to him, inexplicable change in his ordinary mental state, if we understand aright his brief observation at the commencement of the diary, that "the propensity and self-love of his work was passed away, which he himself wondered at;" and that his "inclination for women so hastily terminated, which had been his ruling passion."

At the first he appears to have been quite conscious of the nature and personal origin of his dreams, as well as of their being governed by his dominant thoughts ; but in accordance, it may be said, with his customary habits of thinking, he regarded them in a mystical sense, and applied them to the subjects he wrote on, or studied, or to his religious state as a sinner. Yielding to, as it were, and fostering thus his dreams, they presently became habitual to him, and occasionally passed into the state of actual visions, that is to say of waking dreams,—*hallucinations*. There is nothing whatever to show that the hallucinations of his waking moments were not of essentially the same character as the hallucinations he experienced when asleep. The former, indeed, were the culmination of the same mental and sensorial changes which had led to the latter, and their manifestation was coincident with a marked change in Swedenborg's power of thinking. The augmentation of the sensorial disturbance to the pitch of hallucination in waking moments was, in fact, accompanied with indications of mental disorder.

As in sleep the mind yields implicit belief to the sensorial changes which occur in dreaming, so we find Swedenborg yielding without question implicit belief to the reality of his waking visions. Both the sensorial and mental characteristics of dreaming had passed into his waking moments. He no longer distinguished the true nature of his dreams as dreams, and their intimate dependence upon his dominant thoughts, but thenceforth (unconscious of the vast gulf intervening between the premises and the corollary) he looked upon them as divine revelations ; and into this category he swept without reserve all his dreams, even the commonplace, the ambitious, the horrible, and the erotic.

The erotic dreams which, if we err not, have now for the first time come to light, seem to us to prove most conclusively that Swedenborg's mental faculties were, at this period of his life, more or less disturbed, and that he suffered from hallucinations in the strictly scientific sense of the term. His amatory propensities, as well as religious feelings, determined the form of certain of his dreams, and these, in some instances, assumed a highly obscene character. Now, when we find these dreams deliberately recorded from point to point, even in their most filthy details, and put upon the same level in respect to their spiritual significance as the holiest dreams, the conclusion as to the mental state of the writer is obvious, particularly when, as in Swedenborg's case (who was a celibate, by the way,) he had been a man of pure, unblameable, and religious life.

Swedenborg's account of his strong erotic propensities, and the manifestation of these in his dreams, throws a significant light upon his works on "Conjugial Love," and "The Pleasures of Insanity; or, Scortatory Love," as well as upon passages in other of his theological works.

To say that Swedenborg was a lunatic, is to use a term which conveys to a vast majority of persons the notion of entire lack of reason, and so far, as applied to Swedenborg, would give rise to a false impression. Swedenborg was subject to hallucinations, and these hallucinations influenced his thoughts and governed his conduct for many years. Hallucinations which are not recognised as such are incompatible with sound reason; but it does not follow that this defect of reason should be manifested except in connexion with the hallucinations with which it is allied, or the train of thought immediately dependent upon or arising out of the hallucinations.*

It is but just to add that the Editors of the "Dreams" consider that the additional insight afforded by this newly discovered diary into Swedenborg's mental state when he began to see visions and dream dreams, in nowise detracts from the justness of his claim to their supernatural character.† "Exhibiting," they observe, "the transition period in Swedenborg's life, the change from the worldly to the spiritual, these notes are of great value in enabling us to judge of his psychical condition, which they represent to us as extremely agitated, and into which we can thus look more deeply than was before possible." p. vi. They next inquire, whether those who originally made known these dreams are to be considered as friends or enemies of Swedenborg, and appear to incline to the former theory, although, "on the other

---

* "Ces hommes," writes Lelut, of men like Swedenborg, "étaient doués d'une sensibilité, d'une imagination tellement ardente, et les impulsions intérieures qui les poussaient vers un but nécessité par les besoins et les croyances de l'epoque, croyances et besoins qu'ils partageaient plus que personne et dont ils étaient l'expression vivante;* ces impulsions, dis-je; étaient tellement fortes, que les idées auxquelles elles donnaient lieu ne tardaient pas à se convertir en images sensibles, dont ils n'avaient aucun moyen d'apprecier le manque d'objets dans le monde extérieur, et ils se conduisaient en vertu de ces images, comme dans les passions nous nous conduisons en vertu d'impressions presque aussi vives, et qui nous ôtent momentanément tout moyen de comparaison et de choix. Si l'on veut, ce n'étaient pas des fous; mais, c'étaient des hallucinés, comme il n'y en a plus et comme il ne peut plus y en avoir, des hallucinés dont les visions pourraient être appelées les visions de la raison."—*Récherche des Analogies de la Folie et la Raison.*

† We believe that English, French, and German translations of the "Dreams" exist, but the publication of the English translation (prepared by the Swedenborg Society) has been suppressed, and we have been unable to procure copies of the other translations, whether from their having also been suppressed, or from the impossibility or difficulty of getting them in the ordinary course of trade, we cannot say.

---

* See Cousin on Swedenborg, *Cours de l'Hist. de la Philosph. Mod.*, Lect. xii.

hand, it may seem that he who takes away the wall of a man's bedroom and exhibits him in his nightdress, when he believes himself alone with the God he worships, and before whom he pours forth the inmost thoughts of his heart with earnest supplications for help in his conflicts with an inherited nature, and all the assaults which every sincere Christian understands from the experience of himself and others, can scarcely be the friend of the person thus exposed." p. vii.

"What, then," they ask, "properly is this book, or what does it contain?"

"It is," say these, his apologists, "a journal, or rather a *night-book,* in which Swedenborg noted down his dreams at a time when he seems to have been in a state of inward temptations and conflicts, through which his spirit passed as a sort of fermentation for purification and preparation for his important commission, to receive from the God of heaven and to bring down to the people of the earth, the sublime and pregnant truths which should, according to the prophecies of the apocalypse, at that time be announced and disseminated. Of such a condition the "natural man, which receiveth not the things of the Spirit of God," has no idea. Therefore he who is only superficially educated, who knows not, much less walks in, the way of the new birth, where the soul of man is transformed from a corrupt to an ennobled being, is easily led to pass the doom of condemnation on this work. By such a person it will be, even with reference to its orthography, regarded as the production of an ignorant fool, who has not learned to write his own mother tongue; and who has at one time made subjects of scruples, desires which most other men consider quite natural, and do not think of overcoming, but rather hasten to satisfy ; and at another, has seen in these dreams meanings and indications which could not have any sort of reality, and were therefore mere products of the imagination. A certain class of so-called cultivated people little knows what change the Swedish tongue has undergone within the last century, and still less do such people understand the imagery, the sublimely symbolical forms through which a spiritual world, by means of dreams and visions, under certain circumstances and for definite objects, reveals itself and condescends to a natural world. But not merely the superficially cultivated, but likewise those, who in our days correspond to the scribes and pharisees of the earliest christian epoch, misunderstand and misinterpret a writing like this. Therefore it was stated in a letter from Upsala, written shortly before this book was printed: 'now among Swedenborg's opponents, a manuscript is triumphantly spoken of, which is said to have been found in Westeräs, and from which they believe it appears that his visions proceeded from sensual passions, because he acknowledges therein that he has had severe conflicts of this kind!' But they forget, in drawing such conclusions, that it is through conflicts that victories are won. There is in the entire of this book no certain proof that in these conflicts he *fell*. He speaks, indeed, of himself as a great sinner, for he knows that sin lies even in the desire of anything which removes man from God and heaven; he reproaches himself, and therefore the least thought of self-contentment, or of his own worth, which many another would believe to be more praiseworthy than censurable, and would never think of disapproving in himself. But Swedenborg wishes that the Lord alone should be praised and honoured for all. Therefore, when he explains his own dreams as reprehending what has taken place in his thoughts, he speaks of his unworthiness of all the grace which is shown him, and prays with the deepest humility to be able to abandon himself and everything that is his, in order completely to belong to the Lord alone, and to be unboundedly and without any reserve submitted to his will," p. ix.

We may be wrong in our estimate of the nature of Swedenborg's dreams and visions: but before admitting his claims to divine illumination, we think that confirmatory evidence at least equal to that upon which our faith in the illumination, inspiration, or Divine guidance, of those holy men who wrote the Sacred Scriptures is founded, should be forthcoming.

---

